# Co-Infection and Ventilator-Associated Pneumonia in Critically Ill COVID-19 Patients Requiring Mechanical Ventilation: A Retrospective Cohort Study

**DOI:** 10.3390/biomedicines10081952

**Published:** 2022-08-11

**Authors:** Benjamine Sarton, Marion Grare, Fanny Vardon-Bounes, Anna Gaubert, Stein Silva, Laure Crognier, Béatrice Riu, Thierry Seguin, Bernard Georges, Vincent Minville, Stéphanie Ruiz

**Affiliations:** 1 Réanimation Hôpital Purpan, Centre Hospitalier Universitaire de Toulouse, 31059 Toulouse, France; 2 Plateau Technique Infectiologie, Laboratoire de Bactériologie-Hygiène, Centre Hospitalier Universitaire de Toulouse, 31059 Toulouse, France; 3 INSERM-INRA-ENVT-UPS: UMR1220, Institut de Recherche en Santé Digestive (IRSD), 31059 Toulouse, France; 4 Réanimation Hôpital Rangueil, Centre Hospitalier Universitaire de Toulouse, 31059 Toulouse, France; 5 Département Anesthésie Réanimation, Centre Hospitalier Universitaire de Toulouse, 31059 Toulouse, France

**Keywords:** COVID-19, SARS-CoV-2, intensive care unit, ventilator-associated pneumonia, co-infection, influenza, H1N1

## Abstract

Considering virus-related and drug-induced immunocompromised status of critically ill COVID-19 patients, we hypothesize that these patients would more frequently develop ventilator-associated pneumonia (VAP) than patients with ARDS from other viral causes. We conducted a retrospective observational study in two intensive care units (ICUs) from France, between 2017 and 2020. We compared bacterial co-infection at ICU admission and throughout the disease course of two retrospective longitudinally sampled groups of critically ill patients, who were admitted to ICU for either H1N1 or SARS-CoV-2 respiratory infection and depicted moderate-to-severe ARDS criteria upon admission. Sixty patients in the H1N1 group and 65 in the COVID-19 group were included in the study. Bacterial co-infection at the endotracheal intubation time was diagnosed in 33% of H1N1 and 16% COVID-19 patients (*p* = 0.08). The VAP incidence per 100 days of mechanical ventilation was 3.4 (2.2–5.2) in the H1N1 group and 7.2 (5.3–9.6) in the COVID-19 group (*p* < 0.004). The HR to develop VAP was of 2.33 (1.34–4.04) higher in the COVID-19 group (*p* = 0.002). Ten percent of H1N1 patients and 30% of the COVID-19 patients had a second episode of VAP (*p* = 0.013). COVID-19 patients have fewer bacterial co-infections upon admission, but the incidence of secondary infections increased faster in this group compared to H1N1 patients.

## 1. Introduction

Respiratory viral infections predispose patients to bacterial co-infections and these lead to increased disease severity and mortality [1,2]. However, despite the proven role of bacterial co-infection in the outcome of viral respiratory infections, there is no or low-level evidence regarding either the severe COVID-19 patient’s co-infection upon admission or bacterial secondary infection during the patient’s hospitalization [3]. It is worth noting that despite its potential major value in terms of improvement of prevention and treatment of ventilator-associated pneumonia (VAP) within COVID-19 pandemic planning, there is no available data evidence on the additional risk of bacterial co-infection across the time in these patients, compared to other severely ill cases of respiratory viral infection. Taking into account the growing evidence of the virus-related and drug-induced (e.g., glucocorticoids use) prolonged immunocompromised status of critically ill COVID-19 patients, we hypothesize that these patients will more frequently develop VAP than patients with ARDS from other viral causes and that these patients will have higher rates of VAP recurrence.

Herein, we compared bacterial co-infection at ICU admission and throughout the disease course of two retrospective longitudinally sampled groups of critically ill patients who were admitted to the ICU for either H1N1 or SARS-CoV-2 respiratory infection and depicted moderate-to-severe ARDS criteria upon admission. In both groups, the same diagnosis algorithms of VAP were used, including culture-independent techniques, capable of identifying complex mixed infections without the previous target selection. 

## 2. Materials and Methods

We conducted a retrospective observational study in two ICUs in France, between 2017 and 2020. Patients were screened through the PMSI database (Programme de Médicalisation des Systèmes d’Information) of each participating hospital, using the International Classification of Disease (ICD)-9 code “J09” (viral pneumonia), “J108” (influenza) and “J128” (COVID-19). All medical records were reviewed by investigators including two intensivists (BS, AG). Litigious cases were assessed by an independent adjudication committee formed by an intensivist, an infectiologist and a microbiologist. The ethical committee of University Teaching Hospital of Toulouse approved the study and waived the requirement for informed consent.

### 2.1. Patients

Patients were included if they fulfilled the following criteria: ICU admission for ARDS, (moderate to severe as defined by the Berlin definition (PaO_2_/FiO_2_ ratio < 200) [4], receiving invasive mechanical ventilation (IMV) for more than 48 h before the enrolment, proven respiratory viral infection: SARS-CoV-2 (defined as positive RT-PCR test for SARS-CoV-2 from endotracheal aspiration sample) or H1N1 (defined as positive test RT-PCR or viral culture for H1N1 from endotracheal aspiration sample). Exclusion criteria were: age younger than 18 years, decision to withhold life-sustaining treatment, severe chronic respiratory disease requiring long-term oxygen therapy or home mechanical ventilation, bone marrow transplantation or chemotherapy-induced neutropenia.

### 2.2. Design

We designed a study to compare the incidence rate of VAP between two homogenous groups of ARDS patients related to two independent viral aetiologies (COVID-19 and H1N1). Patients were treated following international guidelines. The incidence rate of VAP per 100 patient-days of IMV and the cumulative probability of VAP at day 28 from the onset of IMV were compared between COVID and H1N1 groups. In addition, the occurrence of VAP recurrence and superinfection in both groups (please see below for definitions) was described. Finally, detailed description of microbiological findings for each ARDS patient’s group was provided.

### 2.3. Diagnosis of Ventilator-Associated Pneumonia

Suspected ventilator-associated pneumonia (VAP) was defined as the presence of new or persistent radiographic features suggesting pneumonia without any other obvious cause and with two of the following: fever > 38 °C, leukocytosis (>11.0 × 10^9^/L) or leukopenia (<3.5 × 10^9^/L), purulent endotracheal aspirate and increasing oxygen requirements. Bronchoalveolar lavage (BAL) was performed in the affected region of the lung identified on thoracic CT scan or chest radiograph. When PaO_2_ was lower than 80, protected minibronchoalveolar lavage could be performed. VAP was diagnosed when a quantitative culture of BAL fluid grew at least one bacterial organism in a concentration ≥ 10^4^ colony forming units (CFU)/mL or when mini-BAL fluid grew at least one bacterial organism in a concentration ≥ 10^3^ CFU/mL. Standard bacteriological culture was coupled with a semi-quantitative multiplex molecular assay (FilmArray Pneumonia Panel Plus (Biofire)-Biomérieux).

### 2.4. Definitions

Appropriate empiric antibiotic therapy was defined as in vitro susceptibility to at least one antibiotic of the organism(s) recovered from the BAL or the minibronchoalveolar lavage samples. In patients with clinical and radiographic evidence of deterioration after initial improvement after a first VAP, “recurrent” infection was suspected if the organism found initially was identified and “superinfection” if a different organism was found. Among recurrent infection we distinguished persistence or relapse VAP cases, according to the diagnostic time and the end of the first antibiotherapy (before and after the end of antibiotherapy, respectively). Pulmonary co-infections were defined as concomitant bacterial pneumonia at patient ICU admission. Early onset VAP complicating ARDS was defined as pneumonia diagnosed between the third and the sixth days after ARDS onset. Late-onset VAP complicating ARDS was defined as pneumonia diagnosed more than six days after ARDS. Recurrent VAPs were successively described (up to third VAP). Patients were monitored for VAP until their discharge from the ICU.

Isolated pathogenic bacteria were described taking into account bacteria species and antibiotics resistance profiles. Multiresistant bacteria were defined as follows: ticarcillin-resistant *P. aeruginosa*, *A. baumannii*, or *S. maltophilia*; extended-spectrum betalactamase-producing Enterobacteriaceae; and methicillin-resistant *S. aureus*. Furthermore, we have defined aspergillosis as probable according to the criteria of EORTC/MSG revised definitions of invasive fungal disease [5].

### 2.5. Data Collection

Demographic data were collected. Baseline health status was described by using a validate scale [6]. Ventilator settings, physiological variables, laboratory data, radiological findings and relevant therapeutic interventions were recorded at ICU admission and across ICU stay for 28 days. We recorded the culture results, antibiotics used, duration of mechanical ventilation and ICU-stay length. Throughout the ICU stay and until weaning off the mechanical ventilator for patients without VAP or the diagnosis of VAP for patients with VAP, we recorded the following factors potentially associated with VAP: enteral nutrition, stress ulcer prophylaxis, neuromuscular blocking agent (NMBA) use during the first 48 h, tracheostomy, emergency reintubation, transport out of the ICU (CT-scan, operating room), prone positioning and renal replacement therapy.

### 2.6. Statistical Analysis

We report means (+/− standard deviation SD), relative risks with 95% confidence intervals (CIs) and medians with interquartile ranges (IQR) as appropriate. Differences between groups were assessed by using the Student’s *t* test, Wilcoxon test, X^2^ analysis or Fisher Exact test. The incidence rate of VAP was calculated as the number of first episodes of VAP divided by the cumulative number of days of intubation for the patients without VAP and up to the date of the first episode of VAP for the other patients. A 95% confidence interval was estimated for the whole cohort and each ARDS viral aetiology group (COVID-19 and H1N1). We used the Z-score test to compare these groups. Kaplan–Meier curves were constructed to assess the cumulative probability of VAP across the time and assess the time from enrolment to death and to unassisted breathing within 30 days. In addition, to investigate the association between VAP incidence and ARDS viral aetiology group, a regression model was built using a Cox proportional-hazards method. To analyse the effect of VAP on ICU mortality, we reported crude ICU mortality. All reported *p* values are two-sided, and the statistically significant threshold was *p* < 0.05. All statistical analyses were performed using R, version 3.6.3 (R Core Team (2022)). R: A language and environment for statistical computing. R Foundation for Statistical Computing, Vienna, Austria. URL https://www.R-project.org/, accessed on 1 April 2020.

## 3. Results

### 3.1. Study Population

**Figure 1:**
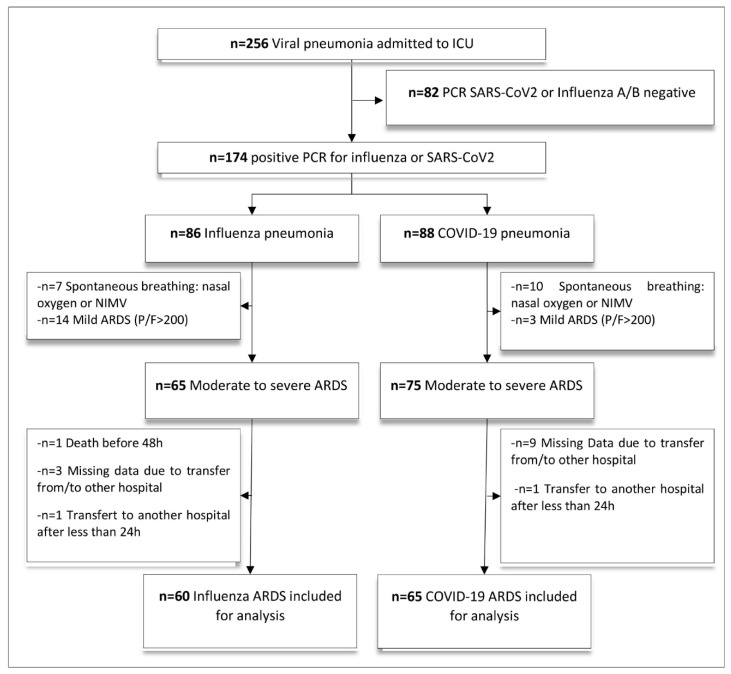
Flow chart of the study. 256 patients with viral pneumonia were admitted in ICU between December 2017 and April 2020. After applying inclusion and exclusion criteria, 60 confirmed H1N1-ARDS and 65 confirmed COVID-19-ARDS were included for analysis. Abbreviations: ARDS = acute respiratory distress syndrome; ICU = intensive care unit; NIMV = non-invasive mechanical ventilation.

From December 2017 to March 2020, 256 patients were admitted to recruiting centres for virus-induced ARDS (Figure 1). Among them, 174 were related to either H1N1 or SARS-CoV-2 proven infection. No simultaneous case of H1N1 and SARS-CoV-2 was identified. After applying inclusion and exclusion criteria, overall, 125 patients were included in the study (60 and 65, in H1N1 and COVID-19 groups, respectively). Table 1 and Supplementary Table S1 report the main characteristics of the 125 patients. The ICU mortality rate was 23% (18 of 125) with no difference between the groups. At ICU admission, median SAPS-II score was 37 (IQR 31–43) and SOFA score was 7 (IQR 6–9). The median PaO_2_/FiO_2_ ratio was 137 (IQR 107–168) and pulmonary compliance was 40 (IQR 31–49) mL/cm H_2_O. We found no clinically significant differences between the H1N1 and COVID-19 groups regarding the main baseline characteristics, co-morbidities or severity score at ICU admission, except for a significant proportion of older patients and diabetes in the COVID-19 group.

### 3.2. Incidence of Ventilator-Associated Pneumonia

**Figure 2:**
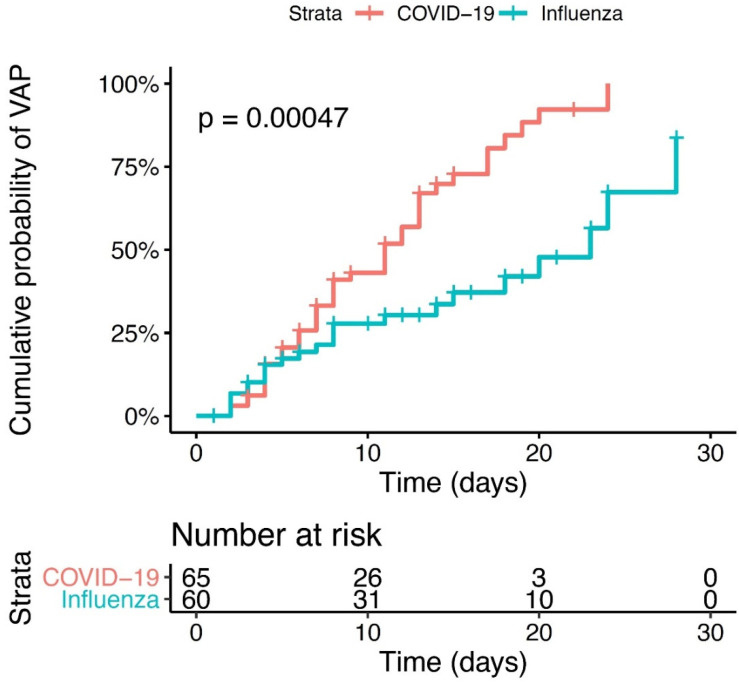
Cumulative probability of VAP. Kaplan Meyer survival analysis from intubation to day 28. Patient with COVID-19-ARDS (red line) or H1N1-ARDS (blue line). *p* < 0.0047.

During the study period, a bacterial VAP was diagnosed in 66 (52%) patients, with 24 cases of early onset and 42 of late onset first VAP episode. The VAP incidence per 100 days of mechanical ventilation was 3.4 (2.2–5.2) in the H1N1 group and 7.2 (5.3–9.6) in the COVID-19 group (*p* < 0.004) (Table 2). Figure 2 shows the cumulative probability of developing bacterial VAP for each virus group. The HR to develop VAP was 2.33 (1.34–4.04) higher in the COVID-19 group compared to H1N1 group (*p* value = 0.002). Bacterial co-infection at the endotracheal intubation time was diagnosed in 20/60 (33%) of H1N1 and 11/65 (16%) COVID-19 patients (*p* = 0.08). A first episode of VAP was identified in 23/60 (36%) H1N1 patients and 43/65 (66%) COVID-19 patients (*p* < 0.003). The cumulative probability to develop a first VAP episode was higher in the COVID-19 group compared to H1N1 group (*p* < 0.0004). We found no group difference between the median times between the onset of invasive mechanical ventilation and the first VAP (7 (IQR 4–14) and 8 (IQR 5–13) days in H1N1 and COVID-19 groups, respectively (*p* = 0.6)). Six of the 60 H1N1 (10%) and 20 of the 65 (30%) COVID-19 patients had a second episode of VAP (*p* = 0.013), whereas 1/60 (1%) of H1N1 and 8/65 (12%) of COVID-19 had three episodes. No episodes of persistent infection were identified in H1N1 patients, but two cases were diagnosed as relapses and five as superinfections in this group. Concerning COVID-19 patients, 7 of 20 (30%) recurrent VAP episodes were diagnosed as persistent, and 13 and 4 of them had a relapse and superinfection VAP, respectively (Table 2). 

### 3.3. Microbiological Findings

**Figure 3:**
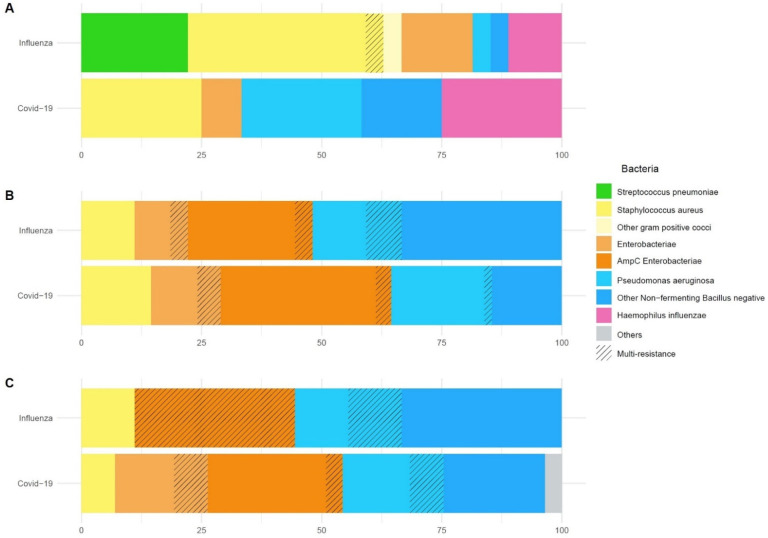
Microbiological findings. Repartition of bacterial strains in percentage of all species isolated. From the top down: (**A**) co-infection at baseline, (**B**) first VAP, (**C**) second and third VAP. For each pathogen group, proportion of multi-drug resistant bacteria appears striped.

Overall, 31 bacteria strains grew in significant concentration in BAL or mini-BAL specimens, which were obtained at the patient’s endotracheal intubation (i.e., co-infections). As indicated in Figure 3 and Table 3, the most common initial co-infection bacteria in H1N1 group were *Staphylococcus aureus* (40%) and *Streptococcus pneumoniae* (22%). COVID-19 bacterial co-infection was still represented by *Staphylococcus aureus* (25%) and *Haemophilus influenzae* (25%). However, in the COVID-19 group, nonfermenting gram-negative bacilli (*Pseudomonas aeruginosa, Stenotrophomonas maltophila, Acinetobacter baumannii*) (41%) and Enterobacteriaceae (8%) were also frequently responsible for bacterial co-infections upon ICU admission.

Regarding early and late onset VAP episodes, Enterobacteriaceae were the most frequently observed bacteria in both groups. For example, during the first VAP episode, Enterobacteriaceae were identified in 10/22 (45%) of H1N1 and 24/43 (55%) of COVID-19 patients. Table 3 reports a detailed description of microbiological findings across patients’ ICU stay. Multidrug resistant bacteria were frequently observed during early and late-onset VAP in both groups (Table 3).

### 3.4. Clinical Outcomes

Of the 125 virus-induced ARDS with VAP, 23 (14%) died in ICU. We observed no significant ICU mortality difference between H1N1 and COVID-19 groups (Supplementary Figure S1. The length of ICU stay was 20 days (IQR 13–30), with no differences between the two groups. As indicated in Table 2, the number of days under invasive mechanical ventilation were similar in H1N1 and COVID-19 groups (13 (IQR 8–21) for H1N1 and 14 (IQR 8–22) for COVID-19 patients).

Empirical antibiotherapy at the patient’s ICU admission (i.e., bacterial co-infection) was adequate in 75% (15/20) and 36% (4/11) of H1N1 and COVID-19 cases, respectively. Antibiotic was initially administered in 100% of H1N1 and COVID-19 cases, respectively (see Table 2). The mean duration of antibiotic empiric treatment was 7 (IQR 5–8.5) days and 5 (IQR 4–6) in H1N1 and COVID-19 groups, respectively. The total number of days under antibiotic treatment (encompassing antibiotherapy duration of co-infection, first VAP and recurrence) were 10 (IQR 7–17) days and 16 (IQR 7–21) days for H1N1 and COVID-19 groups, respectively (*p* = 0.05).

## 4. Discussion

Influenza and SARS-CoV-2 viruses induce severe lung disease requiring intensive care management. However, the clinical features, especially bacterial respiratory co-infections, appear to be radically different between these groups. Patients admitted for COVID-19 to the ICU had fewer bacterial co-infections upon ICU admission but were more likely to develop ventilator-associated pneumonia than patients admitted for influenza.

We found that bacterial co-infection was an important complication in both critically ill H1N1 and COVID-19 patients. In line with previous reports, bacterial co-infection upon ICU admission was diagnosed in almost one third of H1N1 patients [1,7,8,9,10]. As previously reported [11,12,13], co-infection was identified in a lesser proportion of COVID-19 patients at ICU admission. However, our results reveal that this proportion significantly increased across the time in the COVID-19 group (VAP incidence per 100 days of mechanical ventilation: 3.4 (IQR 2.2–5.2) vs. 7.2 (IQR 5.3–9.6) in H1N1 and COVID-19 group, respectively; *p* < 0.004). Overall, the relative risk of developing VAP was 2.33 higher in COVID-19 than H1N1 patients (*p* = 0.002). The groups of patients admitted for influenza or COVID were roughly comparable in terms of patient’s co-morbidities, disease severity and clinical management, hence we suggest that our results are in favour of a specific viral and host factors pathological relationship. It has been suggested that influenza viral infection contributes to respiratory epithelial cell dysfunction and death through disruption of protein synthesis and induction of apoptosis [2]. SARS-CoV-2 infection seems to have similar effects on lower respiratory tract epithelium [14] but also appears to induce profound host immunity disturbances (depletion in the number of effector immune cells and severe T cell and monocyte dysfunction) that could explain the increasing incidence of VAP that we observed across the time in the COVID-19 group [3,15,16,17,18]. 

This SARS-CoV-2 triggered aberrant immune response might also permit the explanation of the significantly higher rate of recurrent infection (identification of the same copathogens as during the first VAP) that we observed in the COVID-19 group, compared to the H1N1 group. Conversely, in H1N1 cases, a different copathogen from the first VAP (i.e., superinfection) was responsible for all the cases of additional VAP throughout the study.

In contrast with the available literature, we reported a detailed description of pathogens that were responsible of co-infection in these two groups of virus-induced ARDS groups. Regarding co-infection upon ICU admission, *Staphylococcus aureus* and *Haemophilus influenzae* were the most frequently isolated copathogens in both groups. These results are in line with previous reports [10,12,13,19,20,21] and support the pathogenesis idea of initial co-infections from the bacteria that colonize the nasopharynx and that can lead to lung co-infection during periods of high respiratory viral shedding [1]. Nevertheless, opposite to previous reports [13,22,23] and French current guidelines for initial empirical antibiotherapy in severe COVID-19, we found neither initial *Streptococcus pneumoniae* co-infection nor intracellular bacteria (e.g., *Legionella pneumophila* or *Mycoplasma pneumoniae)* [24]. Interestingly, concerning late-onset VAPs, we reported a significantly higher proportion of Enterobacteriaceae, more particularly ampC-producing Enterobacteriaceae, in COVID-19 compared to H1N1 patients. Among Non-Fermenting Gram-Negative Bacilli (NFGNB), *P. aeruginosa* was most frequently isolated in COVID-19 patients (13/22). It should be noted that these unexpected microbiological findings in the COVID-19 group have a significant impact in terms of empirical antibiotherapy appropriateness, both during initial co-infection (inappropriate antibiotherapy: 63% vs. 25%, COVID-19 vs. H1N1 respectively; *p* = 0.05) and first VAP (inappropriate antibiotherapy: 13% vs. 8%, COVID-19 vs. H1N1 respectively; *p* = 0.03). In case of VAP recurrence, an initial inappropriate empirical antibiotherapy was only observed in COVID-19 patients. However, antibiotic treatment was adapted in a second time in all the cases, taking into account respiratory sampling. It can be argued that this high rate of VAP recurrence is related to COVID-19 related immunocompromised status and the microbiological challenge that is represented by the treatment of the pathogens that were observed during the 2nd and 3rd VAP cases (AmpC-producing Enterobacteriaceae and *P. aeruginosa*) [25].

Despite the higher incidence of VAP in COVID-19 patients throughout the study, we found no difference in terms of mortality between viral groups (28-day mortality: 18% and 10% for H1N1 and COVID-19, respectively), ICU or in-hospital length of stay. Similar outcomes have been previously reported in the literature [11,26]. We did not observe any excess mortality at D28 in the COVID group compared with patients with influenza, in contrast to the work of Nseir et al. [27].

To the extent of our knowledge, there are few reports that have compared the bacterial co-infection rates between H1N1 and COVID-19 ARDS patients. However, these studies were exclusively focused on a highly selected patient group under ECMO or on the first episode of ventilator-associated lower respiratory tract infections [28,29]. One additional strength of our work was a detailed description of our microbiological findings. Nevertheless, our study design is not without limitations: future studies should focus on larger and longer, prospective longitudinal studies that will specifically explore the impact of bacterial co-infection in the COVID-19 pandemic.

In summary, we reported a higher incidence of ICU acquired bacterial VAP in COVID-19 compared to H1N1 ARDS patients. Clearly, COVID-19 patients have fewer bacterial co-infections upon admission, but the incidence of secondary infections increased faster in this group compared to H1N1 patients. Alongside this result, a higher rate of VAP recurrence associated to an unexpected incidence of ampC-producing Enterobacteriaceae, NFGNB and *P. aeruginosa* in COVID-19 patients suggests a long-lasting immune-paralysis phenomenon in patients infected by SARS-CoV-2. We think that our study provided valuable data on the bacterial pathogens causing co-infections, secondary infections and antimicrobial resistance in this challenging setting, thereby helping inform antibiotic prescribing policy.

## Figures and Tables

**Table 1:** Demographics and patient’s characteristics at ICU admission.

	Total (n = 125)	Influenza (n = 60)	COVID-19 (n = 65)	*p*
General characteristics				
Age (years)	62 (55–69)	60 (52–65)	65 (56–72)	0.022
Male gender	92 (74)	39 (65)	53 (82)	0.058
BMI (kg/m^2^)	27 (24–31)	27 (23–32)	27 (25–31)	0.663
Heath status				
Good	70 (56)	37 (61)	33 (50)	0.015
Mild to moderate	22 (17)	9 (15)	13 (20)	
Serious	25 (20)	6 (10)	19 (29)	
Severe	3 (2)	3 (5)	0 (0)	
Blood group				
A/B/AB	52/81 (64)	21/41 (51)	31/40 (77)	0.025
O	38/81 (46)	20/41 (49)	9/40 (22)	
Underlying medical condition				
Diabetes	21 (16)	6 (9)	15 (25)	0.034
Dyslipidaemia	25 (20)	13 (20)	12 (20)	1.000
Hypertension	45 (31)	14(21)	31 (51)	0.893
Myocardial infarction	16 (12)	9 (15)	7 (10)	0.660
Heart failure	3 (2)	3 (4)	0 (0)	0.104
COPD	15 (12)	10 (15)	5 (7)	0.192
Asthma	6 (4)	2 (3)	4 (6)	0.681
Smoker	34 (27)	18 (30)	16 (24)	0.398
Chronic kidney disease	10 (8)	2 (3)	8 (12)	0.098
Immunocompromised	11 (8)	7 (11)	4 (6)	0.440
Solid tumour	15 (12)	7 (11)	8 (12)	1.000
Non-steroidal anti inflammatory	12 (9)	6 (10)	6 (9)	1.000
Steroid use	10 (8)	8 (13)	2 (3)	0.041
ACE inhibitors or ARB treatment	38 (30)	17 (28)	21 (32)	0.820
Characteristics at ICU admission				
SAPS II	37 (31–43)	36 (31–46)	37 (32–42)	0.748
SOFA	7 (6–9)	8 (6–9)	7 (5–8)	0.003
Glasgow coma scale	15 (15–15)	15 (15–15)	15 (15–15)	0.330
Mean blood pressure (mmHg)	64 (60–69)	65 (58–71)	63 (60–66)	0.475
Norepinephrine administration	67 (53)	51 (85)	16 (65)	<0.001
Acute renal failure				
K-DIGO 0–1	94 (75)	42 (69)	52 (79)	0.023
K-DIGO 2–3	31(25)	18 (30)	13 (21)	
PaO_2_/FiO_2_ (mmHg)	137 (107–168)	138 (103–158)	136 (109–175)	0.168
PEEP applied (cm H_2_O)	10 (8–12)	10 (8–12)	10 (8–12)	0.286
Tidal volume (mL/kg predicted body weight)	6.5 (6.2–7.0)	6.4 (6.0–7.0)	6.7 (6.3–7.0)	0.069
Respiratory system compliance (mL/cm H_2_O)	40 (31–49)	34 (26–41)	46 (38–54)	<0.001

Results are expressed as median (quartiles) or numbers (%). Abbreviations: ACE = angiotensin-converting enzyme; ARB = angiotensin receptor blockers; BMI = body mass index; COPD = chronic obstructive pulmonary disease; K-DIGO = Kidney disease improving global outcomes; NSAI = non-steroidal anti-inflammatory; PEEP = positive end-expiratory pressure; SAPS II = simplified acute physiology score; SOFA = sequential organ-failure assessment.

**Table 2:** Outcomes.

	Total (n = 125)	Influenza (n = 60)	COVID-19 (n = 65)	*p*
Primary outcome				
VAP incidence/100 IMV days (IC 95%)	5.2 (4.1–6.6)	3.4 (2.2–5.2)	7.2 (5.3–9.6)	0.004
Secondary outcomes				
Bacterial co-infection at baseline				
Prevalence	31/125 (24)	20/60 (33)	11/65 (16)	0.055
Empiric antibiotic therapy	125/125 (100)	60/60 (100)	65/65 (100)	1.000
Duration of antibiotic therapy (days)	6 (4–7)	7 (5–8.5)	5 (4–6)	<0.001
Appropriateness of empiric antibiotic therapy				
Appropriate	12/31 (38)	15/20 (75)	4/11 (36)	0.056
Inappropriate	19/31 (61)	5/20 (25)	7/11 (63)	
First VAP				
Prevalence	66/125 (52)	23/60 (38)	43/65 (66)	0.003
Delay (days from intubation)	8 (4–13)	7 (4–14)	8 (5–13)	0.626
Early VAP (≤6 days)	24/66 (36)	11/23 (47)	13/43 (30)	0.251
Late VAP (>7 days)	42/66 (63)	12/23 (52)	30/43 (69)	
Appropriateness of empiric antibiotic therapy				
Appropriate	24/66 (36)	4/23 (17)	20/43 (46)	0.035
Inappropriate	8/66 (12)	2/23 (8)	6/43 (13)	
None	33/66 (50)	16/23 (69)	17/43 (39)	
Appropriate final antibiotic therapy	66/66 (100)	23/23 (100)	43/43 (100)	1.000
Second and third VAP				
Prevalence of 2nd VAP	26/125 (20)	6/60 (10)	20/65 (30)	0.013
Prevalence of 3rd VAP	9/125 (7)	1/60 (1)	8/65 (12)	0.050
Recurrence				0.005
Persistence	11/35 (31)	0/7 (0)	11/28 (39)	
Relapse	15/35 (42)	2/7 (28)	13/28 (46)	
Superinfection	9/35 (25)	5/7 (71)	4/28 (14)	
Appropriateness of empiric antibiotic therapy				
Appropriate	11/35 (31)	2/7 (28)	9/28 (32)	0.393
Inappropriate	7/35 (20)	0/7 (0)	7/28 (25)	
None	17/35 (48)	5/7 (71)	12/28 (42)	
Appropriate final antibiotic therapy	35/35 (100)	7/7 (100)	28/28 (100)	1.000
ICU Stay				
Invasive ventilation, days	14 (8–21)	13 (8–21)	14 (8–22)	0.397
Death at D28	18 (14)	11 (18)	7 (10)	0.342
ICU mortality	23 (18)	13 (21)	10 (15)	0.500
ICU stay, days	20 (13–30)	18 (13–25)	21 (12–31)	0.335
Antibiotic therapy, days	13 (7–19)	10 (7–17)	16 (7–21)	0.051

Results are expressed as median (quartiles) or numbers (%). Prevalence, delay of occurrence and therapeutic management are reported for baseline co-infection, first VAP and second/third VAP. All data are censored at day 28. Abbreviations: ICU = intensive care unit; IMV = invasive mechanical ventilation; VAP = ventilator associated pneumonia.

**Table 3:** Microbiological findings.

	Influenza (n = 60)	COVID-19 (n = 65)
Bacterial co-infection at baseline	n = 20	n = 11
Species identified in respiratory samples (total number)	27	12
Staphylococcus aureus	11 (40)	3 (25)
Streptococcus pneumoniae	6 (22)	0 (0)
Hemophilus influenzae	3 (11)	3 (25)
Enterobacteriaceae	4 (14)	1 (8)
AmpC Producing β-lactamase Enterobacteriaceae	0 (0)	0 (0)
Non fermenting gram-negative bacilli	2 (7)	5 (41)
Pseudomonas aeruginosa	1 (3)	3 (25)
Others	1 (3)	0 (0)
Multi-drug resistant species	1 (5)	0 (0)
Methicillin-resistant Staphylococcus Aureus	1	0
First VAP	n = 22	n = 43
Species identified in respiratory samples (total number)	26	62
Staphylococcus aureus	3 (11)	9 (14)
Enterobacteriaceae	10 (36)	31 (50)
AmpC producing β-lactamase Enterobacteriaceae	7 (26)	22 (35)
Non fermenting gram-negative bacilli	13 (50)	22 (35)
Pseudomonas aeruginosa	5 (19)	13 (20)
Acinetobacter baumannii	1 (3)	7 (11)
Stenotrophomonas maltophilia	7 (53)	2 (3)
Others	0 (0)	0 (0)
Multi-drug resistant species	4 (14)	6 (9)
Extended spectrum β-lactamase	1	3
Enterobacteriaceae cephalosporinase	1	2
Pseudomonas aeruginosa Ceftazidime-R or Carbapenem-R	2	1
Second and third VAP	n = 8	n = 28
Species identified in respiratory samples (total number)	9	57
Staphylococcus aureus	1 (11)	4 (7)
Enterobacteriaceae	3 (33)	27 (47)
AmpC producing β-lactamase Enterobacteriaceae	3 (33)	16 (28)
Non fermenting gram-negative bacilli	5 (55)	25 (43)
Pseudomonas aeruginosa	2 (22)	12 (21)
Acinetobacter baumannii	2 (22)	5 (8)
Stenotrophomonas maltophilia	1 (11)	8 (14)
Others	0 (0)	1 (1)
Multi-drug resistant species	4 (36)	10 (17)
Extended spectrum β-lactamase	0	4
Enterobacteriaceae cephalosporinase	3	2
Pseudomonas aeruginosa Ceftazidime-R or Carbapenem-R	1	4
Fungal pneumonia		
Probable pulmonary aspergillosis	8 (13)	6 (9)

Abbreviation: Ceftazidime-R or Carbapenem-R = resistance to ceftazidime or carbapems.

## Data Availability

The datasets used and/or analysed during the current study are available from the corresponding author on reasonable request.

## Supplementary Materials

The following supporting information can be downloaded at: https://www.mdpi.com/article/10.3390/biomedicines10081952/s1, Table S1: Risk factors of ventilator associated pneumonia. Underlying condition and ICU management considered as potential risk factors of VAP. Abbreviations: COPD = chronic obstructive pulmonary disease; ECMO = extracorporeal membrane oxygenation; PPI = proton pump inhibitors. Figure S1: Cumulative probability of death. Kaplan Meyer survival analysis from intubation to day 28. Patient with COVID-19-ARDS (red line) or H1N1-ARDS (blue line).
