# Supplementary material for: Co-Infection and Ventilator-Associated Pneumonia in Critically Ill COVID-19 Patients Requiring Mechanical Ventilation: A Retrospective Cohort Study
Source: Biomedicines. 2022 Aug 11;10(8):1952. doi: 10.3390/biomedicines10081952 (PMC9405710; doi:10.3390/biomedicines10081952)

Figure S1: Cumulative probability of death. Kaplan Meyer survival analysis from intubation to day 28. Patient with COVID-19-ARDS (red line) or H1N1-ARDS (blue line).

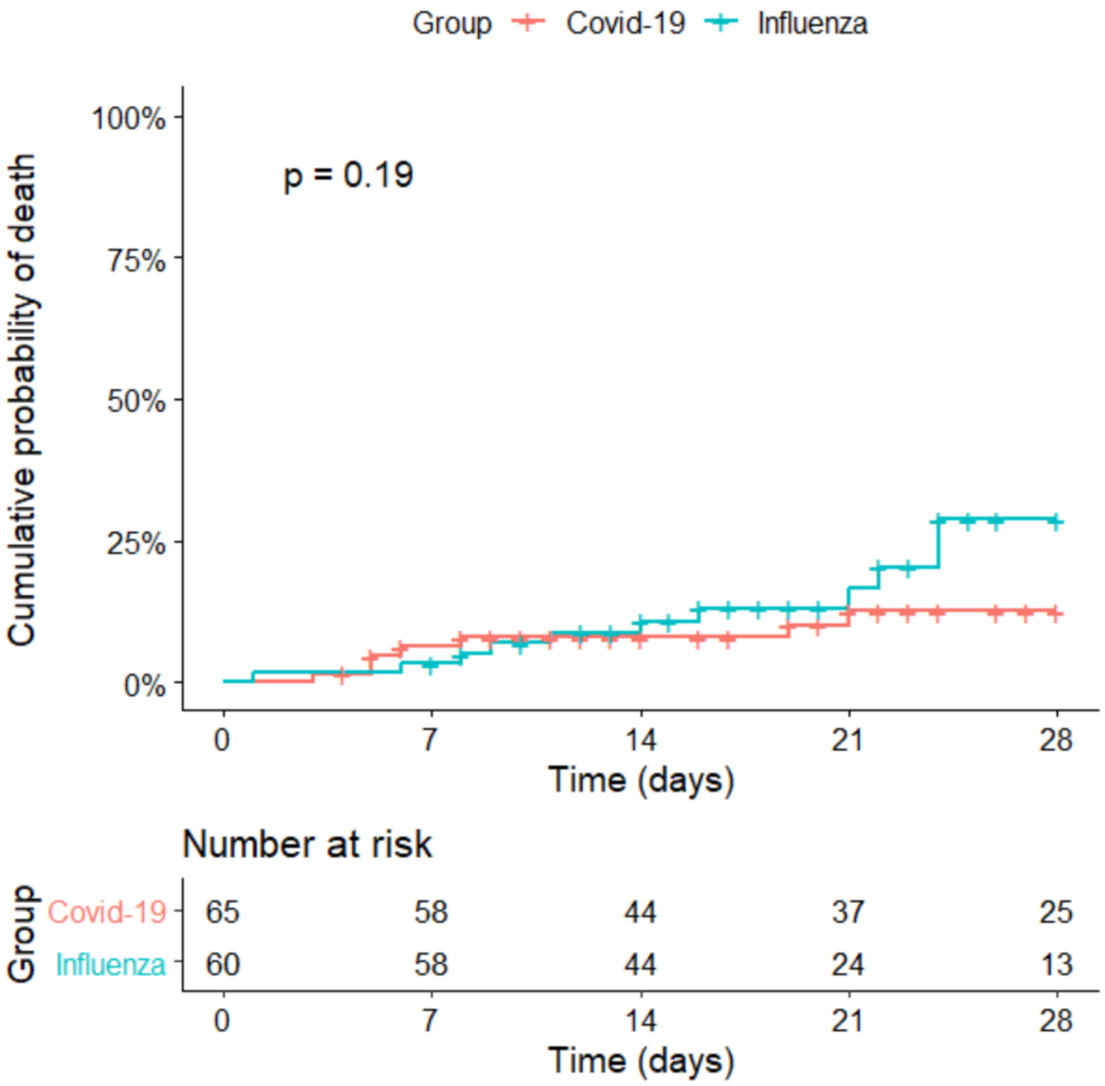

Supplement: Supplementary file 1 [file biomedicines-10-01952-s001.zip › Figure S1 Cumulative probability of death.pdf]
