# Supplementary material for: Co-Infection and Ventilator-Associated Pneumonia in Critically Ill COVID-19 Patients Requiring Mechanical Ventilation: A Retrospective Cohort Study
Source: Biomedicines. 2022 Aug 11;10(8):1952. doi: 10.3390/biomedicines10081952 (PMC9405710; doi:10.3390/biomedicines10081952)
Supplement: Supplementary file 1 [file biomedicines-10-01952-s001.zip › Table S1 SOM_1_VAP-riskFactors.pdf]

Table S1: Risk factors of ventilator associated pneumonia.

	Total (n = 125)	Influenza (n=60)	COVID-19 (n = 65)	*p*
Demographics				
Male gender	92 (74)	39 (65)	53 (82)	0.058
Underlying medical conditions				
Diabetes	21 (16)	6 (9)	15 (25)	0.034
COPD	15 (12)	10 (15)	5 (7)	0.192
Immunocompromised	11 (8)	7 (11)	4 (6)	0.440
Solid tumour	15 (12)	7 (11)	8 (12)	1.000
Characteristics at baseline				
SAPS II[c]	37 [31 – 43]	36 [31 – 46]	37 [32 – 42]	0.748
SOFA[d]	7 [6 – 9]	8 [6 – 9]	7 [5 – 8]	0.003
PaO2/FiO2 (mmHg)	137 [107 – 168]	138 [103 – 158]	136 [109 – 175]	0.168
Glasgow coma scale	15 [15 – 15]	15 [15 – 15]	15 [15 – 15]	0.330
ICU management				
Invasive mechanical ventilation, days	14 [8 – 21]	13 [8 – 21]	14 [8 – 22]	0.397
Prone positioning	62 (49)	18 (30)	44 (73)	< 0.001
Tracheostomy	21 (15)	4 (3)	17 (26)	0.007
ECMO	19 (15)	15 (25)	4 (6)	0.007
Muscle blockade use	110 (88)	53 (88)	57 (87)	1.000
Antibiotic therapy at admission	125 (100)	60 (100)	65(100)	1.000
Stress ulcer prophylaxis (PPI)	71 (56)	35 (58)	39 (60)	0.994
Selective digestive decontamination	0 (0%)	0(0%)	0(0%)	1.000

Underlying condition and ICU management considered as potential risk factors of AVP.
Abbreviations: COPD= chronic obstructive pulmonary disease; ECMO= extracorporeal membrane oxygenation; PPI= proton pump inhibitors.
